# A Tissue-Specific Approach to the Analysis of Metabolic Changes in *Caenorhabditis elegans*

**DOI:** 10.1371/journal.pone.0028417

**Published:** 2011-12-05

**Authors:** Jürgen Hench, Ivana Bratić Hench, Claire Pujol, Sabine Ipsen, Susanne Brodesser, Arnaud Mourier, Markus Tolnay, Stephan Frank, Aleksandra Trifunović

**Affiliations:** 1 Department of Neuropathology, Institute of Pathology, University Hospital Basel, Basel, Switzerland; 2 Department of Laboratory Medicine, Division of Metabolic Diseases, Karolinska Institutet, Stockholm, Sweden; 3 Cologne Excellence Cluster on Cellular Stress Responses in Aging-Associated Diseases (CECAD), Institute for Genetics, University of Cologne, Cologne, Germany; 4 Lipidomics Core Facility, CECAD and Institute for Medical Microbiology, Immunology, and Hygiene, Cologne, Germany; 5 Max-Planck-Institut for Biology of Ageing, Cologne, Germany; University of Pennsylvannia, United States of America

## Abstract

The majority of metabolic principles are evolutionarily conserved from nematodes to humans. *Caenorhabditis elegans* has widely accelerated the discovery of new genes important to maintain organismic metabolic homeostasis. Various methods exist to assess the metabolic state in worms, yet they often require large animal numbers and tend to be performed as bulk analyses of whole worm homogenates, thereby largely precluding a detailed studies of metabolic changes in specific worm tissues. Here, we have adapted well-established histochemical methods for the use on *C. elegans* fresh frozen sections and demonstrate their validity for analyses of morphological and metabolic changes on tissue level in wild type and various mutant strains. We show how the worm presents on hematoxylin and eosin (H&E) stained sections and demonstrate their usefulness in monitoring and the identification of morphological abnormalities. In addition, we demonstrate how Oil-Red-O staining on frozen worm cross-sections permits quantification of lipid storage, avoiding the artifact-prone fixation and permeabilization procedures of traditional whole-mount protocols. We also adjusted standard enzymatic stains for respiratory chain subunits (NADH, SDH, and COX) to monitor metabolic states of various *C. elegans* tissues. In summary, the protocols presented here provide technical guidance to obtain robust, reproducible and quantifiable tissue-specific data on worm morphology as well as carbohydrate, lipid and mitochondrial energy metabolism that cannot be obtained through traditional biochemical bulk analyses of worm homogenates. Furthermore, analysis of worm cross-sections overcomes the common problem with quantification in three-dimensional whole-mount specimens.

## Introduction

Basic metabolic principles exhibit a remarkable degree of evolutionary conservation. While many key regulators of metabolism have been identified through biochemical and molecular approaches using mammalian model systems, invertebrate genetic model systems including the nematode *C. elegans* have widely accelerated the discovery of genes essential for the maintenance of an organism's metabolic homeostasis. Thus, a number of genes involved in the regulation of lipid synthesis and storage, mitochondrial function and insulin signaling have been identified using *C. elegans* as a model system. Many of these genes are described as important modifiers of lifespan in *C. elegans,* probably by regulating metabolic shifts during reproduction and aging [1]. So far, various methods have been developed to assess metabolic changes in worms [2]–[5]. Unfortunately, they tend to be performed in a bulk manner where whole worm homogenates are analyzed, precluding analyses and understanding of metabolic changes in specific tissues. As a consequence, even highly significant changes in a specific tissue or organ are often under-represented or masked in such bulk analyses, highlighting the need for refinement of existing methods.

The use of conventional histological stains in whole-mount *C. elegans* specimens and sectioning of worms has rarely been reported [3], [4], [6]–[8]. Our results indicate that sectional histology can be applied to define novel phenotypes in *C. elegans.* Here, we demonstrate the usefulness of fresh frozen serial sections combined with enzyme histochemistry and other staining methods to analyze worm metabolism. We also present data revealing novel links between various genetic backgrounds and metabolic states via their histological fingerprint on tissue and organism level. These protocols are intended to serve as a diagnostic toolbox to provide a comprehensive picture of the metabolic state of an individual worm and will, as we believe, be beneficial for future research efforts of the “worm community” aimed at understanding metabolic changes in *C. elegans.*

## Results

### Worm Histopathology – Usefulness of Classical Stains

**Figure 1:**
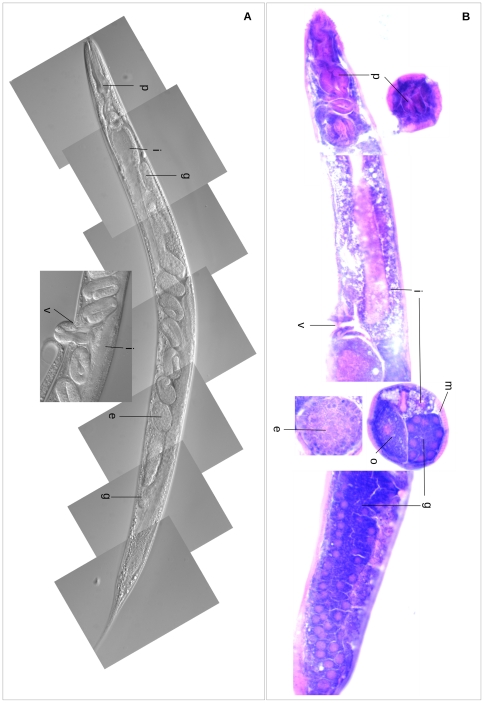
*C. elegans* morphology visualized by H&E staining. Comparison of DIC images (A) and H&E sections (B) of adult wild type worms at day 1 of adulthood. p-pharynx, v-vulva, e-embryo, g-germline, m-body wall, o-oocyte, i-intestine.

Worm anatomy is often imaged in the sagittal plane of the semitransparent body of *C. elegans* obtained by differential interference contrast microscopy (DIC, Figure 1A) and, more rarely, by transmission electron microscopy (TEM). We believe that fresh frozen sections contribute relevant data that cannot be obtained by classical “whole-mount” preparations: (I) they retain the original morphology (which is often a problem with classical mounting of stained *C. elegans*); (II) they also retain the enzymatic activity, antigenicity, lipids and carbohydrates, and thus can be used for biochemical as well as immunological analyses, and (III) antibodies and histochemicals can easily penetrate cells and tissues.

**Figure 2:**
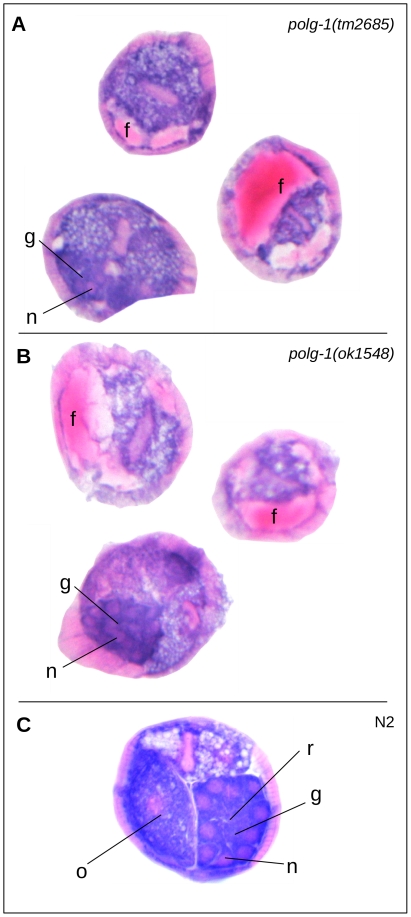
Pathomorphology in *polg-1(tm2685)* and *polg-1(ok1548)* mutants visualized by H&E staining. Morphology of *polg-1(tm2685)* (A), *polg-1(ok1548)* (B) mutants and wild type - N2 (C) animals in transverse sections through adult worms at day 1 of adulthood. g-distal gonad with n-nuclei and r-rachis, o-germline, f-fluid (“ascites-like”). Note that the nuclei are scattered in the mutant gonad and that there is no rachis. The oocytes are not readily apparent.

Here we show how the worm presents on H&E stained, fresh frozen sections (Figure 1B). We started with conventional H&E staining, as this is the most widely used medical staining technique that gives a good overview of general morphology, and therefore can be used as a reference for other dyes. H&E provides both longitudinal and transverse resolution, the latter being almost impossible to obtain without sectioning (Figure 1B). Furthermore, different tissues display specific patterns of hematoxylin and eosin binding, allowing pathomorphological features to become readily visible (Figure 1 and 2).

We have previously described molecular characteristics and phenotype of the mitochondrial DNA polymerase gamma deficient *C. elegans* mutants, *polg-1(ok1548)* and *polg-1(tm2685)* [9], [10]. Mutant worms are characterized by almost complete sterility and premature death from organ protrusion through the vulva. We have now assessed the morphology of these mutants at day 1 of adulthood by H&E histology. Cross sections of homozygous *polg-1(tm2685)* and *polg-1(ok1548)* mutant worms reveal two striking changes when compared to wild type animals (Figure 2A-C). (I) We observe accumulation of an eosinophilic mass in the pseudocoelomic space, histomorphologically resembling an effusion of protein-rich fluid, comparable to e.g. intraalveolar lung edema, or ascites in humans. This eosinophilic fluid did not stain with either PAS or Oil-Red-O, indicating that it does not contain high molecular weight carbohydrates or lipids (data not shown). (II) The disorganization of the distal gonad is visualized on cross-sections, replicating our previous data based on TEM and fluorescent microscopy 3D reconstruction (Figure 2A-C) [9].

**Figure 3:**
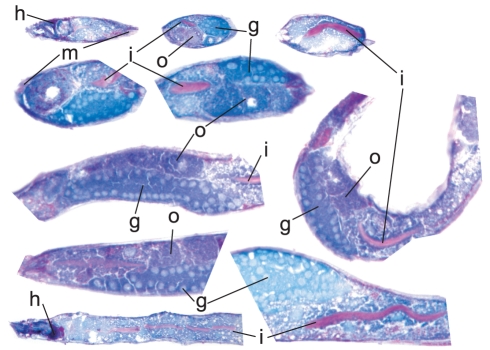
Detection of carbohydrates by periodic acid-Schiff's (PAS) staining in wild type animals (N2). High molecular weight carbohydrates are stained in red. The blue hematoxylin counter-stain visualizes the organ structures: g-gonad, o-oocytes, m-body wall, i-intestine, h-head. There is a pronounced carbohydrate content in the head around the pharynx, in the intestinal lumen as well as in some of the gut granules. Empty vesicles within the gut represent lipid droplets.

In addition to H&E, worm sections were stained with periodic acid Schiff's (PAS) reagent which is commonly used to detect carbohydrates in tissues. The PAS reagent labels a fraction of the gut granules, the intestinal lumen lined by a glycocalyx, and structures within the head of the worm, reflecting accumulation of high molecular weight carbohydrates (Figure 3) [11].

### Oil-Red-O Staining of Neutral Fat Deposits

**Figure 4:**
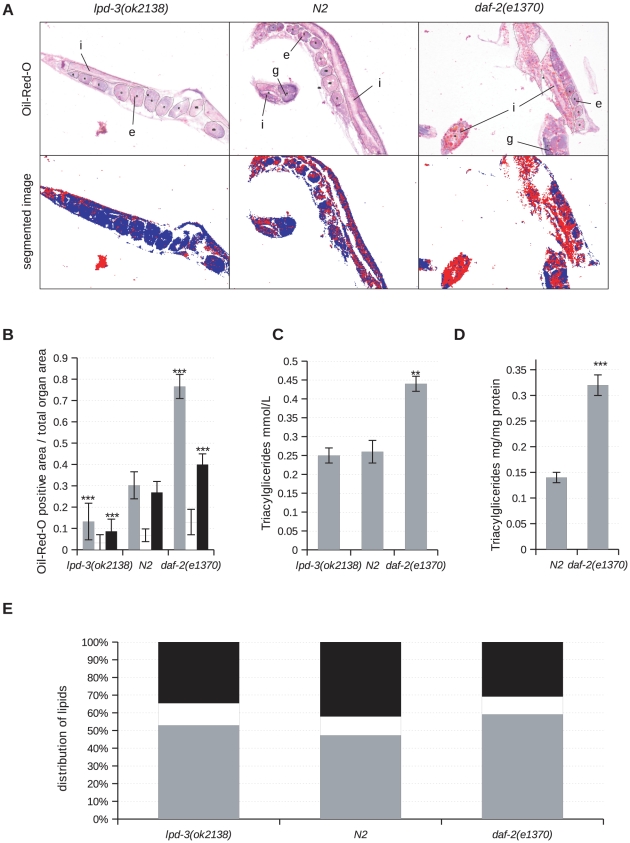
Lipid content determined by Oil-Red-O staining of frozen sections of *lpd-3(ok2138),* N2 and *daf-2(e1370)* animals. (A) Image analysis upon Oil-Red-O staining. Upper row represents annotated areas of respected tissues for analysis. Lower row indicates background-subtracted, color-segmented images (red = Oil-Red-O; blue = hematoxylin) of corresponding mutants. e-embryo, g-germline, i-intestine. Lipid level (B) and lipid distribution (E) in intestine (gray), distal (white) and proximal gonad arm with intrauterine embryos and oocytes (black). Bars represent the 95% confidence interval with upper and lower limits. Triacylglyceride level measured by colorimetry (C) and by thin-layer chromatography (D) in *lpd-3(ok2138),* N2 and *daf-2(e1370)* animals. Bars represent standard error of the mean. All animals were collected at day 1 of adulthood from synchronized populations. Asterisks indicate statistical significance in comparison to wild type (Student's t-test, ** p<0.01, *** p<0.001).

Oil-Red-O stains neutral fat deposits in frozen sections and is applicable to whole-mount *C. elegans* samples [3]. Color image segmentation of Oil-Red-O stained and hematoxylin counter-stained sections results in reproducible, proportional data that reflect overall lipid content in individual animals (Figure 4A). This approach is easily extended by defining regions of interest (ROIs) to assess lipid content in a subset of tissues. Our data confirm the presence of high lipid content in intestinal cells and the proximal germline of the wild type (N2 Bristol), as well as a substantial fraction of lipids contained in embryos of gravid hermaphrodites, whereas only low lipid content is found in the distal germline (Figure 4A, B, E).

To further validate our method, we analyzed the lipid content and spatial distribution in *daf-2 (e1370)* mutants known to accumulate high levels of lipids (Figure 4A, B) [2]. Our data confirm that *daf-2(e1370)* mutants have a two-fold increase in intestinal lipid content compared to wild type worms. A smaller increase in lipid content is also observed in embryos, distal and proximal gonads (Figure 4B). Similarly, we analyzed lipid content and distribution in the *lpd-3(ok2138)* mutants (Figure 4A-B). LPD-3 (lipid depleted) is expressed prominently in the intestine, a major site of fat storage [12]. Its activity is required for normal lipid metabolism. Animals that have reduced *lpd-3* activity as a result of RNA-mediated gene interference are paler and skinnier than wild type animals and lack lipid storage granules in the intestine [13], [14]. Our results demonstrate that *lpd-3(ok2138)* mutants contain approximately 50% of the wild type lipid levels in all examined tissues (intestine, distal and proximal gonad arm with embryos) (Figure 4B). Interestingly, our data also indicate that the proportion of fat stored within the largest tissues of the worm, *i.e.* intestine and gonad, remains largely unchanged irrespective of overall lipid content (Figure 4E). Within the gonad, the proximal arm containing oocytes and early intrauterine embryos stains more densely in contrast to the proliferating distal gonad arm (Figure 4B, E). We correlated our data with results obtained by biochemical colorimetric analysis of triacylglycerides (TAG) as well as by TAG thin-layer chromatography (TLC, Figure 4C, D). Both analyses showed a two-fold increase in TAG levels in *daf-2*(*e1370*) mutants. Additionally, the validity of our TLC method was confirmed by measuring the lipid content of *daf-2(e1370)* dauer larvae. As anticipated, we have detected a ten-fold increase in TAG levels in day 1 *daf-2(e1370)* dauer larvae compared to wild type day 1 adults, which is in agreement with previous results (data not shown) [15]. The colorimetric analysis did not detect a lower lipid content in *lpd-3(ok2138)* animals. Unfortunately, due to technical difficulties inherent in reaching sufficient amounts of *lpd-3(ok2138)* mutants, we were unable to perform TLC analyses on this strain.

We have also compared our results obtained by Oil-Red-O staining of fresh frozen sections with data obtained on the same strains (N2, *daf-2(e1370)* and *lpd-3(ok2138)*) that were fixed and permeabilized prior to staining as a whole-mount (Figure S1A). It becomes easily apparent that sectioning improves, or rather simplifies overview of lipid distribution compared to examination of the entire three-dimensional specimen where the observer or analytic machinery has to deal with light scattering and absorption from staining outside the focal plane. Further, we have analyzed worms that were fixed and stained prior to sectioning (Figure S1B-C). The lipid staining in this case appears blurred and is difficult to analyze. In summary, comparative analysis of all applied variants of Oil-Red-O staining (i.e. whole-mounts, sections after permeabilization and native fresh frozen sections stained with Oil-Red-O), convincingly demonstrates that morphology and resolution are best preserved in fresh frozen sections that were natively stained with Oil-Red-O (Figure S2).

### Assessing Respiratory Complex Activities

**Figure 5:**
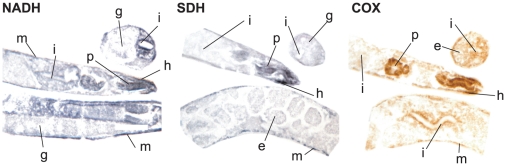
NADH, SDH and COX staining on fresh frozen sections of wild type animals (N2). Sectioning was performed on synchronized populations at day 1 of adulthood. m-body wall, i-intestine, p-pharynx, h-head portion of body wall, g-germline, e-embryo.

**Figure 6:**
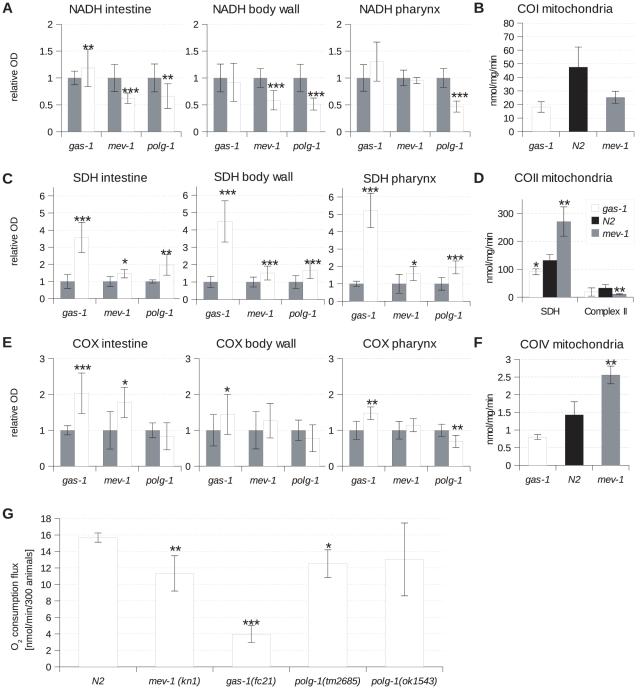
Analysis of mitochondrial function in *C. elegans* by histochemistry in *gas-1(fc21)*, *mev-1(kn1)* and *polg-1(ok1548)* animals. NADH (A), SDH (C) and COX (E) activity in intestine, body wall, and pharynx tissue. Gray bars refer to wild type animals (N2) and white bars to corresponding mutant animals indicated on the chart. (B) Complex I, (D) Complex II and SDH, (F) Complex IV activity measured on isolated mitochondria by spectrophotometry. (G) Oxygen consumption flux of young adults at day 1 of adulthood. Bars represent standard deviation of the mean. Asterisks denote statistical significance in comparison to wild type (Student's t-test, * p<0.05, ** p<0.01, *** p<0.001).

So far, a limited number of studies have addressed the consequences of different mutations on the activity of the mitochondrial respiratory chain complexes [16]–[19]. Previous studies mainly assessed mitochondrial function by evaluation of either respiration of whole animals *in vivo* or enzyme activity of uncoupled sub-mitochondrial particles *in vitro* [16], [20]–[22]. We aimed to examine mitochondrial function on the level of single tissues and therefore adapted methods to visualize and quantify those changes *in situ*. Activities of the electron transport chain (ETC) complexes were measured after histochemical staining for different enzymatic activities on fresh frozen *C. elegans* sections (Figure 5 and 6). We performed an extensive number of control reactions for the histochemical staining to exclude any unspecific reactivity. Absence of staining in fresh frozen sections either by substrate-less solution, electrophilic dye solution alone or by specific inhibitors of ETC complexes I, II and IV (rotenone, malonate and potassium cyanide respectively) confirmed the specificity of the staining reactions (data not shown). The underlying biochemical reactions provided linear correlation between optical density of the detection system and enzymatic activity [23]. We recorded the change in optical density during the staining reaction over time (Figure S3). As expected, exponential staining increase takes place after a specific period of time (NADH after 5 min, SDH after 10 min, COX after 2 h; all at 20°C). Reactions for quantification were inspected in tight intervals and terminated within the exponential phase. We have performed quantitative assessment of complex I (NADH:ubiquinone oxidoreductase, EC 1.6.5.3), complex II (succinate dehydrogenase, SDH, EC 1.3.5.1), and complex IV (cytochrome c oxidase, COX, EC 1.9.3.1) activity (Figure 6A, C, E). Our data clearly show that the ETC activities between individual tissues differ significantly (Figure 5 and 6). All three measured ETC complexes are highly active in the pharynx, body wall and in the apical portions of intestinal cells of wild type worms. In contrast, only minor activity is detected in the germline (Figure 5). Corresponding organs in developing embryos within gravid hermaphrodites exhibit a drastically lower respiratory activity than the respective organs of their parents, indicating modulation of the metabolic profile during development (data not shown). Although cross and longitudinal sections do not separate the body wall well enough into muscle and hypodermal cells, diagonal cuts reveal a striped pattern upon enzymatic staining. This pattern is characteristic for *C. elegans* muscle cells (Figure S5). Thus, staining intensity of the body wall appears to mainly correspond to enzymatic activity of body wall muscles, not hypodermis.

Strains carrying mutations in protein subunits of either complex I (*gas-1(fc21))* or complex II (*mev-1(kn1))* of the ETC were tested against wild type and the corresponding differences were determined on a single tissue level. We also included the *polg-1(ok1548)* mutant strain in these studies, as the loss of POLG-1 leads to mtDNA depletion and therefore general decrease in the level of ETC complexes in mutant worms [9].

In order to study how the changes in ETC activities influence oxygen consumption in these mutants, we also performed high-resolution respirometry on young adult worms. We found a significantly lower level of oxygen consumption in *mev-1(kn1)*, *gas-1(fc21)* and *polg-1(tm2685)* mutants. Oxygen consumption of *polg-1(ok1548)* mutants was also decreased compared to wild type worms, although this reduction did not reach statistical difference (Figure 6G).

In parallel, we analyzed activities of respiratory chain complexes in isolated sub-mitochondrial particles by spectrophotometry. In the *gas-1(fc21)* mutant this analysis revealed decreased complex I, complex II, and IV activities (Figure 6B, D, F). However, these changes were not statistically significant. Interestingly, in all analyzed tissues, histochemical staining shows that the NADH activity is basically unchanged whereas complex II and IV activity is clearly increased in *gas-1(fc21)* mutant (Figure 6A, C, E). The discrepancy between enzyme activity assessed on isolated mitochondria and the NADH activity measured on fresh frozen sections could reflect an increase in respiratory chain content *in vivo* in order to compensate for the complex I defect.

Complex II consists of four subunits (SDHA-D) needed to channel electrons coming from the oxidation of succinate to fumarate, to ubiquinone. In addition, two peripheral subunits (SDHA-B) act as the enzyme succinate dehydrogenase of the tricarboxylic acid cycle (TCA). The *mev-1(kn1)* mutant carries a mutation in one of the two anchoring subunits of the succinate dehydrogenase located in the inner mitochondrial membrane, therefore affecting primarily complex II activity (SDH activity able to reduce quinone). In agreement with the decrease in respiration observed in *mev-1(kn1)* worms (Figure 6G), we observed a significant decrease in the complex II activity in isolated submitochondrial particles (Figure 6D). However, the total SDH activity in this mutant is clearly increased as shown by both, *in situ* enzyme histochemistry and *in vitro* activity measurements (Figure 6C, D). Interestingly, it seems that changes in the ETC enzyme activities of major tissues prevail in the measurements *in vitro*. For example, we have detected a decrease in the NADH dehydrogenase activity and an increase in complex IV activity *in vitro* (Figure 6B, F). This correlates very well with the *in situ* enzyme histochemistry in the intestine of *mev-1(kn1),* where similar changes were observed (Figure 6A, E). Overall, our quantitative histochemical data from *mev-1(kn1)* and *gas-1(fc21)* correlated well with the respective spectrophotometric results.

We further analyzed the *polg-1(ok1548)* mutant whose gonad is particularly depleted of mitochondria [9], [10]. Although somatic tissues functionally appear unaffected by the mutation [9], we find a reduction in NADH dehydrogenase activity in all examined tissues (intestine, body wall and pharynx) (Figure 6A). Previously, we described decreased transcript levels of NADH dehydrogenase subunit 5 most likely reflected by the decreased complex I activity of these mutants [9]. In contrast, SDH activity in *polg-1(ok1548)* was generally increased, perhaps reflecting compensatory upregulation (Figure 6A, C). The COX activity in *polg-1(ok1548)* is significantly lower only in the pharynx and has a tendency to decrease in the body wall and intestine (Figure 6E). Collectively, these findings suggest that, even though one would expect an overall mitochondrial defect due to general mtDNA depletion, the effect on metabolism still remains tissue-specific in analogy to mammalian systems.

### Immunohistochemistry with Hematoxylin Counter-Staining

**Figure 7:**
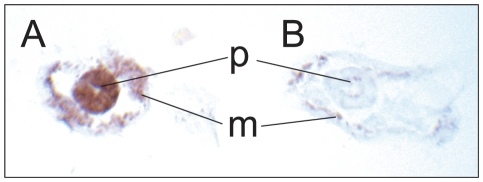
GFP detection in *polg-1(ok1548)/mIn1* animals with DAB immunodetection system. *polg-1(ok1548)/mIn1* animals carrying a GFP-labeled balancer chromosome with pharyngeal muscle and gut expression, analyzed at day 1 of adulthood in frozen sections. Transverse sections through head. (A) GFP-positive pharynx, i.e. heterozygous or wild type for *polg-1(ok1548).* (B) GFP-negative pharynx, *i.e.* homozygous for *polg-1(ok1548).* m- body wall, p*-*pharynx. Anti-GFP specifically labels GFP-expressing tissues.

Antibody-based histochemical stains with various detection systems have been applied in *C. elegans* [24]. While most of that work has been performed in worm whole-mounts, we find that procedures used for mammalian tissue sections can be applied to frozen *C. elegans* sections, too. Immunohistochemistry in *C. elegans* research could enable the detection of proteins whose fluorescent properties unfortunately get lost during almost all histochemical procedures. Here we demonstrated that an antibody against green fluorescent protein (GFP) clearly distinguishes genotypes in a mixed population of a balanced mutant strain (*polg-1(ok1548)/mIn1*, GFP expression in the pharynx and to a lesser extent in the intestine, Figure 7) [25]. Prior to tissue block casting, automated worm sorting was applied, and anti-GFP immunohistochemistry confirmed the sorting efficiency [26]. In situations where automated sorting equipment is unavailable or cannot be applied, tissue blocks containing a mixed population of GFP-positive and -negative animals can alternatively be analyzed through serial sections. Individual outlines of the worms and their positions in the block allow three to four longitudinal serial sections to be obtained (each 7 µm in thickness) from adult individuals (data not shown).

## Discussion

Here we demonstrate the usefulness of classical histochemistry in *C. elegans* sections that to our knowledge has only marginally been applied in the *C. elegans* research field. In contrast, histochemistry is a standard procedure for many other model organisms, and an important part of pathomorphological diagnostics of human diseases.

Based on H&E morphology, we can now describe a novel phenotype characterized by pseudocoelomic fluid accumulation. We interpret this pathological change as the underlying morphological correlate of the previously described lethal organ protrusion observed in polymerase gamma mutants [9], [10]. In analogy to human pathophysiology we suggest the term "ascites-like" for this phenotype. Most strikingly, two different alleles of the *polg-1* gene show this ascites-like phenotype that had so far gone undetected. We favor the hypothesis that this fluid accumulation results from a somatic malfunction in the worm caused by mtDNA depletion mainly in the excretory system. An alternative explanation might be a gonad malfunction. Yet, as the ascites-like phenotype can already be observed at day 1 of adulthood when *polg-1* mutants are still able to produce several (inviable) embryos, we consider this second scenario less likely. Regardless of the definitive nature of this particular phenotype, we do believe that H&E screening for pseudocoelomic fluid accumulation may be particularly useful in screening for excretory cell-related phenotypes.

Furthermore, we have tested the PAS stain in *C. elegans* sections and find that it robustly labels known carbohydrate-rich areas of the animal such as the intestinal glycocalyx. In addition to this prominent structure, there is a reproducible PAS-positive area in the head, around the pharynx. The PAS reaction can be extended by carbohydrate digestion steps allowing a more precise determination of the carbohydrate type (e.g. diastase-PAS to digest glycogen with amylase [8]).

Studies of lipid metabolism in *C. elegans* are increasingly coming into focus. *C. elegans* fat stores are dynamic in terms of their size and number, being subject to developmental processes or environmental changes [27]. Major fat storage is represented by TAG stored in lipid droplets and yolk, matching our data. Lipid droplets, located mostly in the intestine and in small fractions in the hypodermis, are cellular organelles surrounded by a monolayer of phospholipids and proteins [28]. Yolk is produced in the intestinal cells, secreted into the body cavity from where it is taken up by oocytes and subsequently transported intracellularly by members of the vitellogenin protein family [13], [29]. TAGs present the main energy storage in *C. elegans.* They are synthesized through esterification of various saturated, monosaturated and polysaturated fatty acids obtained from bacterial diet or synthesized *de novo* from acetyl-CoA and glycerol. Until now, several methods have been used for assaying TAG storage in worms. These methods can be divided into compound separation, labeling, and detection of physical lipid properties. Chemical analysis of *C. elegans* lipids is mostly based on chloroform-methanol extraction where lipids are separated either by thin-layer or solid-phase-exchange chromatography [30]. TAG storage can also be analyzed by enzymatic assays [31]. These approaches are readily applicable and can provide data on fatty acid composition and quantity. However, they are often limited by the large numbers of age-synchronized animals needed for lipid extraction. For example, synchronized day 1 adults of *daf-2(e1370)* have a two-fold increase in TAG levels compared to wild type animals prepared in the same way, while this difference is lost when mixed-age populations of *daf-2(e1370)* and N2 were analyzed by TLC (Figure S4).

Labeling methods measure lipid content by staining of either fixed or live worms. Fixed worms can be stained by lipophilic dyes such as Sudan Black or Oil-Red-O. The major disadvantage is that the required cell permeabilization and fixation makes this method prone to artifacts [3], [5]. Previous studies have shown that Oil-Red-O staining correlates with TAG levels examined by solid-phase chromatography followed by gas chromatography and mass spectrometry [3], [32]. The precision of Oil-Red-O stain for quantification of lipid content has been subject to debate because Oil-Red-O does not stain all fat stores in worms, and suboptimal quantification algorithms are being used [3], [5] In addition, artifacts may also be introduced by fixation, permeabilization, and washing steps. Nevertheless, the Oil-Red-O stain allows to visualize fat levels more accurately than other lipophilic dyes [3]. For example, Nile Red is a vital lipophilic dye often used in live worms. However, it was recently shown that Nile Red does not stain the complete lipid stores in worms, but rather accumulates in gut granules that are lysosome-related organelles [2], [3], [5]. Use of BODIPY, another vital dye often used to stain fat stores in worms was also questioned with respect to the variable efficiency in dye uptake related to changes in food consumption [32]. Physical properties of lipids have also been studied with anti-stokes Raman scattering microscopy, a label-free method for visualizing all fats [5], [27]. This advanced method, abbreviated CARS, is based on physical properties of lipid molecules [33]. The advantage is that CARS does not require staining or fixation of the animals and that it resolves size and 3D-lipid distribution of lipid stores. Its major limitation is the dependency on highly specialized equipment. In addition, CARS signals exhibit a non-resonant background that limits detection sensitivity and creates imaging artifacts [27], [34]. Recently, a novel method based on stimulated Raman scattering (SRS) microscopy was used to detect fat stores in *C. elegans* [34]. Unfortunately, as in the case of CARS microscopy, a dependency on highly specialized equipment severely limits applicability of SRS microscopy. Ideally, an improved lipid staining method would avoid the fixation step and instead physically open the cells, allowing to measure fat distribution on tissue level and permitting relative quantification of labeled lipids against a tissue size reference. We believe that these prerequisites for an optimal lipid quantification method in worms are met by our sectioning, staining and imaging protocol. However, admittedly, this proposed method also has its limitations, as it does not allow to address the subcellular details of lipid stores in *C. elegans* due to the general limitations of light microscopy. The same limitation applies to the smaller tissues/organs of the animal such as neurons, muscle cells, somatic gonad and to some extent hypodermal cells. These tissues are difficult to annotate at sufficient precision for accurate lipid quantification, while drastic changes would certainly be detectable at high magnification.

In human medicine, the degree of pathological lipid storage (such as in liver steatosis) is routinely assessed by the appearance and size of empty vacuoles within the cells. If there remain questions about the nature of those vacuoles that lose their lipid content during tissue dehydration for paraffin embedding, native or formalin-fixed tissue is analyzed by staining with lipophilic dyes in frozen sections. We applied this well-established method in *C. elegans* in a largely unmodified form and find the results of Oil-Red-O staining convincing. The two major advantages of our protocol in *C. elegans* are the well-defined tissue size reference and the possibility of applying multiple histochemical stains, including anti-GFP immunohistochemistry and enzymatic reactions to a single individual animal. Up to four longitudinal sections can be obtained per individual adult worm, while from larvae (not used in this study) we usually manage to retrieve at least two sections (data not shown). Distinct organ separation based on histomorphology can be achieved by background counter-stains such as hematoxylin. Image analysis is dramatically simplified and improved by avoiding third dimension measurements. ROI selection in image files allows the data to be read out globally or in a tissue-specific manner. There is no more need for arbitrary lipid content units. Two or more samples can be co-stained on a single glass slide, examined and preserved for screening and future reference. In order to validate our method, we analyzed two mutant strains, *daf-2(e1370)* and *lpd-3(ok2138)*. Our data indicate that the lipid levels in *daf-2(e1370)* mutants are two-fold higher than in wild type and thus match data from biochemical TAG content analysis. We also analyzed *lpd-3(ok2138)* mutants and show that these mutants contain half the amount of fat relative to wild type (N2). TLC and enzyme-based colorimetric assays did not detect this decrease in lipid content of *lpd-1(ok2138)* animals. One reason could be that we are unable to determine subtle changes in TAG levels by enzyme-based colorimetry based assays. Alternatively, in addition to TAGs, Oil-Red-O might also stain other neutral lipids. Strikingly, the inter-organ ratio of lipid deposits remains largely unchanged while the overall content changes (Figure .6E, B). We compared various ways of Oil-Red-O staining, in particular fresh frozen sections, fixed whole-mounts, and sectioned fixed whole-worms. We find that Oil-Red-O staining of fresh frozen sections gives by far the highest resolution with well-preserved morphology. However, sectioning of previously fixed and stained animals also simplifies quantification in comparison to whole-mounts.

The accurate biochemical assessment of the OXPHOS system is necessary for the diagnosis and investigation of mitochondrial diseases. Human muscle biopsies are commonly examined by enzyme histochemistry for NADH, SDH and COX activity. This rather direct biochemical detection system is fully validated by the routine use in medical diagnostics. Now, we have applied this diagnostic panel to *C. elegans* fresh frozen sections in order to obtain in-depth insight into mitochondrial function of an individual worm. We have additionally validated applied methods for usage in *C. elegans* with a set of control stainings including, but not limited to, specific single-ETC complex inhibitors.

The tissue-specific staining pattern that was obtained after ETC enzyme histochemistry, matches the distribution of mitochondria as well as the expectations regarding mitochondrial activity within a single worm. We found the highest activity in the pharynx, which is best compared to mammalian heart muscle, also matching the ultra-structurally verified abundance of mitochondria in this organ [35], [36]. Functionally, the pharynx muscle obviously transforms large amounts of energy. The same holds true for body wall muscles where mitochondria are oriented in longitudinal lines [9], and this could be observed in diagonal sections where both muscle and hypodermal cells become distinguishable (Figure S5). The third and last prominence of high enzymatic activity is concentrated at the apical portion of the nematode's enterocytes where resorption of nutrients takes place. While our method is able to separate the enzymatic activities in the intestine, pharynx, body wall, and head, it is limited in distinction between body wall muscles and hypodermal cells. Our data also indicate that individual tissues contribute differentially to the overall respiration rates of the animal.

We have detected a general increase in COX and SDH enzymatic staining in both analyzed mutant strains (*gas-1(fc21)* and *mev-1(kn1)*). A similar upregulation is well described in a number of mitochondrial diseases and generally used as a hallmark of mitochondrial diseases [37]. We believe that the increase in complex II and complex IV activity likely represents a compensatory mechanism, ensuring enough electrons to be shifted along the respiratory chain. We have detected a mild increase in NADH staining in *gas-1(fc21)* animals, and a decrease in *mev-1(kn1)* mutants. The opposing effects could be explained by a different nature of the two mutants affecting complex I and II of the ETC, respectively. Furthermore, one has to bear in mind that the NADH tetrazolium reductase stain used in our protocol is not exclusive for complex I activity. In addition to mitochondria, many components of the inter-myofibrillar network, such as the sarcoplasmic reticulum and T-tubules in mammals are also stained, as they contain NADH-oxidizing enzymes [38]. Likewise, there may be structures in *C. elegans* tissues causing a similar effect.

Functional compensation may lead to increased component synthesis through positive feedback, resulting in increased *in situ* enzymatic activities. Respiratory chain defects often trigger mitochondrial accumulation with an increase of overall mitochondrial activities, possibly including partially defective ones. This may sometimes mask a deficiency, particularly if only partial. Indeed, upregulation of mitochondrial mass and volume was reported previously for the *gas-1(fc21)* mutant [39]. Therefore, the upregulation of ETC activities detected upon staining could just reflect an increase in the mitochondrial mass in mutant worms. This feature will be lost when isolated mitochondria are analyzed *in vitro*.

The measurement of enzyme activities by spectrophotometric methods gives valuable information about enzyme activities of the catalytic component of the various respiratory complexes following disruption of the inner mitochondrial membrane. Unfortunately, no universally agreed standardization exists for these assays or assay conditions. Indeed, a recent program among European laboratories found wide variation in respective assay results on the same samples [40]. Another important limitation is that activities are determined under *in vitro* conditions with addition of artificial substrates, which do not reflect the physiological environment of intact mitochondria. In addition, enzyme histochemistry and spectrophotometric methods are based on different electron acceptors and these also could contribute to differences in data obtained by these two methods. One also has to keep in mind that enzymatic assays performed on isolated mitochondria have the disadvantage that standard isolation methods retrieve only a fraction of the original mitochondrial content. In particular, difficulties with cuticle and tissue disruption in *C. elegans*, combined with a loss of mitochondria during isolation may lead to a selective over-representation of larger organs. Apart from this limitation, for standard enzymatic assays, large amounts of animals are required in order to obtain enough mitochondria.

One mechanistic reason contributing to discrepancies in ETC activity measurements may be the fact that disruption of mitochondrial structure during preparation of submitochondrial particles might dramatically influence ETC complex activities [41]. This is illustrated by the analysis of mice deficient for NDUFS4 subunit of complex I, where NADH activity in submitochondrial particles was basically undetectable by spectrophotometric assays. However, complex I driven oxygen consumption in intact mitochondria was about half that of controls [41], which may be attributed to the disruption of mitochondrial structure, causing loss of complex I activity in these mutants [41]. It is not difficult to envision that, similarly, a mutation in *gas-1* could lead to decreased complex I stability, which in turn could affect the stability of super-complexes, finally impacting the fidelity of spectrophotometric assays.

Although the usefulness of spectrophotometric assays of enzyme activity in the analysis of mitochondrial (dys)function is undeniable, we believe that ETC enzyme histochemistry holds a great advantage for mitochondrial analysis in *C. elegans* as it allows usage of less animals, and permits tissue specific probing with a high level of robustness and accuracy.

In summary, we have transferred well-established histochemical procedures to frozen tissue sections in *C. elegans* and demonstrate their reliability as well as their applicability for mutant screening on a morphological and metabolic level. In addition, our data show that the above-described methods can reliably be used in the monitoring of lipid changes, morphological defects or enzymatic activities on tissue level in *C. elegans*. We developed methods for detecting subtle changes in lipid storage, carbohydrate accumulation and mitochondrial function at *C. elegans* tissue level and hope that the protocols described herein will be instrumental in the establishment of novel links between metabolism-regulating genes and their effects at tissue level.

## Materials and Methods

### Nematode Strains and Cultivation

Wild type (N2, Bristol) and mutant alleles *(daf-2(e1370)*, *mev-1(kn1), gas-1(fc21), lpd-3 (ok2138), polg-1(ok1548), polg-1(ok1548)/mIn1[mIs14[P_pes-1::GFP,P_myo-2::GFP]dpy10 (e128)]II)* were obtained from the *C. elegans* Genetics Center (CGC) and cultivated on standard nematode growth medium (NGM). *polg-1(tm2685) allele* was obtained from the National BioResource Project (Japan). The E. coli strain OP50 served as the sole food source [42]. Synchronized populations were obtained by bleaching of worm strains and starting the culture from the resulting isolated embryos [43]. All histochemical analyses were performed on day 1 of adulthood of hermaphrodite worms. Cultures were processed to tissue blocks on day 1 of adulthood for synchronized cultures unless otherwise stated in the text. In case of heterozygous worm populations, animals were sorted at day 1 of adulthood by a COPAS (Complex Parametric Analyzer and Sorter, Union Biometrica) in order to isolate the desired subpopulation.

### Tissue Blocks

Worms were washed off the NGM plates along with the remaining bacteria using M9 buffer and transferred into 1.5 ml centrifuge tubes (Eppendorf, Germany) [42]. Upon collection, worms were centrifuged at 1300×g for one minute and the supernatant was discarded. Approximately 1 ml layer of TissueTek® carbowax tissue embedding medium (Sakura Fintek, Netherlands) was filled into a fresh 1.5 ml tube and the worm pellet was transferred on top of TissueTek® with a pipette. Attention was exercised to ensure that the worm pellet along with M9 remnants did not exceed a volume of 0.5 ml. The tubes were then centrifuged at 1300×g for five minutes, yielding a worm pellet, which was transferred into a Cryomold® (Sakura Fintek, Netherlands) along with sufficient amounts of carbowax to fill the mold. In order to relax the worms and to make them sediment, the cryo-moulds were incubated at approximately 8°C for 10 min, resulting in an increase in longitudinally oriented animals and decreased stress-induced "curling" of the worms. The blocks were frozen in a glass dish filled with isopentane that had been submerged in liquid nitrogen prior to use, thereby avoiding direct contact between liquid nitrogen and the sample. Frozen blocks were stored at −20°C and cut on a cryo-microtome to produce 7 µm thick sections collected on microscopy glass slides (“Super Frost”, Menzel, Germany) for staining. Each glass slide contained up to three sections of the strains of interest as well as the reference strain.

### Hematoxylin and Eosin Staining (H&E)

H&E (Hämalaun / Erythrosin, Medite, Germany) staining was performed at room temperature with staining times adjusted for *C. elegans.* Sections were fixed in buffered histology grade 4% formalin, washed (H_2_O), stained with hematoxylin (2 min), washed (H_2_O), differentiated in HCl/ethanol, rinsed (H_2_O), turned blue in warm tap water, stained with eosin (1 min), shortly rinsed (H_2_O), dehydrated in ascending ethanol concentrations, transferred to xylenes and finally embedded with Pertex® routine embedding medium (Medite, Germany) under cover slides.

### Periodic Acid Schiff's Staining (PAS)

PAS staining was performed as in routine diagnostic histochemistry for frozen sections. Staining times were adjusted for *C. elegans.* Fresh sections were collected on Super Frost (Menzel, Germany) glass slides, fixed in buffered histology grade 4% formalin, rinsed in H_2_O (dist.), dipped in periodic acid (0.5%), shortly rinsed with H_2_O (dist.), incubated in Schiff's reagent (Merck, Germany) (5 min) and washed in running tap water. Then hematoxylin stain was applied as described for H&E. Sections were dehydrated in ethanol and covered by routine histochemical embedding medium. All the staining steps were performed at room temperature.

### GFP Immunohistochemistry

Anti-GFP immunohistochemistry was performed on frozen *C. elegans* sections using mouse monoclonal IgG_1_ antibody sc-101525 (Santa Cruz Biotechnology, California, USA). Visualization of the primary anti-GFP antibody (dilution from 1∶100 to 1∶200) was done by 3,3′-diaminobenzidine according to the manufacturer's instructions (Peroxidase Substrate Kit, SK-4100, Vector Laboratories, California, USA). Sections were counter-stained with hematoxylin as described for H&E, dehydrated in ethanol and covered by routine histochemical embedding medium.

### Oil-Red-O Staining

Oil-Red-O solution was prepared by dissolving 0.3 g Oil-Red-O (Sigma Aldrich, Switzerland) in 100 ml isopropanol. Sections were natively stained in Oil-Red-O solution (20 min) at room temperature, then rinsed in running tap water (2 min). Hematoxylin counter-staining was performed without differentiation in HCl-ethanol and sections were rinsed (H_2_O). Sections were transferred to H_2_O (dist.) and then embedded with aqueous medium (Medi-Mount, Medite, Germany). Fixation and permeabilization based fat staining with Oil-Red-O in whole worms was performed as described [32]. Upon fixation, permeabilization, and staining, animals were embedded with aqueous medium (Medi Mount, Medite, Germany).

### Quantification of Triglyceride Levels by Thin-Layer Chromatography

Approximately 30'000 worms (5 independent replicates) collected at day 1 of adulthood were homogenized in 1 ml of H_2_O using the Homogenisator Precellys 24 (Peqlab, Germany) at 6'500rpm for 30 s. The protein content of the homogenate was routinely determined using bicinchoninic acid. After addition of 4 ml of methanol and 2 ml of chloroform, lipids were extracted for 24 h at 37°C. The liquid phase was separated by filtration, and the insoluble tissue residues were further extracted for 24 h at 37°C in 6 ml of methanol/chloroform 1∶1 (v/v) and finally in 6 ml of methanol/chloroform 1∶2 (v/v). The extracts were pooled, and the solvent was evaporated in a stream of nitrogen. The residues were purified using a modification of the Bligh-Dyer procedure as previously described [44]. For the determination of the worm lipid content, the lipid extract was applied to 20×10 cm high performance thin layer chromatography (HPTLC) Silica Gel 60 plates (Merck, Germany), which were pre-washed twice with chloroform/methanol 1∶1 (v/v) and air-dried for 30 min. For quantification of triacylglycerols each lane of the TLC plate was loaded with the equivalent of 25 µg. The TLC solvent system used for the detection of triacylglycerols was hexane/toluene 1∶1 (v/v), followed by hexane/diethyl ether/glacial acetic acid 80∶20∶1 (v/v/v). For quantitative analytical TLC determination, increasing amounts of triolein (Sigma Aldrich, Germany) were applied to the TLC plates in addition to the lipid samples. For detection of lipid bands, the TLC plates were sprayed with a phosphoric acid/copper sulfate reagent (15.6 g of CuSO_4_(H_2_O)_5_ and 9.4 ml of H_3_PO_4_ (85 %, w/v) in 100 ml of water) and charred at 180°C for 10 min [45]. Lipid bands were then quantified by densitometry using the TLC-Scanner 3 (CAMAG, Germany) at a wavelength of 595 nm.

### Quantification of Triglyceride Levels by Colorimetry

Approximately 10'000 adult worms (day 1 of adulthood) were collected and washed with M9 buffer. Adult worms were frozen at −80°C and, prior to analysis, were exposed for additional 3 freeze/thaw cycles followed by 3 cycles of sonication for 30 s. Upon sonication, worms debris were precipitated at 1300×g for 1 min at 4°C. Quantification of triglycerides (TAG) was performed according to the manufacturer's instructions (EnzyChrom Triglyceride Assay kit, BioAssay Systems, California, USA). Optical density was read at 570 nm by a plate reader (Paradigm Detection Platform, Beckman Coulter, California, USA). Determined TAG levels were normalized to protein content which was determined by the Bradford method.

### COX Staining

Enzyme histochemical staining for cytochrome oxidase activity (COX) was performed as described [46]. Incubation time and temperature were adjusted for *C. elegans.* Sections were incubated at room temperature for up to 2 h in incubation medium (3,3′ diaminobenzidine tetrahydrochloride 15 mg, cytochrome C (Sigma Aldrich, Switzerland) 30 mg, bovine catalase (Sigma Aldrich, Switzerland) 3 mg, 50 mM phosphate buffer 27 ml, sucrose 2.25 g). Reactions were stopped prior to saturation (continuous visual examination) by washing in H_2_O (dist.), dehydrated in ethanol and covered by routine histochemical embedding medium.

### NADH Staining

Enzyme histochemical staining of nicotinamideadenindinucleotide diaphorase (NADH) activity was performed as described with incubation time and temperature adjusted for *C. elegans* [47]. Sections were incubated under sight at room temperature until one section turned dark blue (3-8 min). The reaction was stopped by fixation in 4% formalin (5 min). Sections were rinsed with H_2_O, transferred to H_2_O (dist.) and embedded with aqueous medium (Medi-Mount, Medite, Germany). Incubation medium: Tris-HCl buffer 0.2 M pH 7.4 30 ml, tetranitroblue tetrazolium (NBT) 30 mg, NADH 24 mg.

### SDH Staining

Enzyme histochemical staining of succinate dehydrogenase (SDH) activity was performed as previously described with incubation time and temperature adjusted for *C. elegans* [47]. Sections were incubated under sight at room temperature until one section turned dark blue (usually 20-30 min). The reaction was stopped by fixation in 4% formalin (5 min). Sections were rinsed with H_2_O, transferred to H_2_O (dist.) and embedded with aqueous medium (Medi-Mount, Medite, Germany). Incubation medium: phosphate buffer 0.07 M 100 ml, sodium succinate 0.2 M 100 ml, tetranitroblue tetrazolium (NBT) solution 0.1% 200 ml, phenazin methosulphate 20 mg.

### Recording of Changes in Optical Density Resulting from NADH, SDH and COX Enzymatic Activities

Staining solutions for NADH, SDH and COX activity were prepared as described above. These staining solutions were applied directly on the fresh frozen sections (7 µm thickness) of N2 animals and NADH, SDH and COX enzymatic activities were recorded over time at 20°C. The reactions were covered with a cover slide and cover slide fragments were used as spacers. Reactions for COX were sealed with hot paraffin wax in order to avoid drying artifacts during the long incubation time. Images were recorded in regular intervals with a monochrome camera (ORCA 2R, Hamamatsu, Japan) mounted on a transmission light microscope (AxioPlan, Zeiss, Germany). The camera was controlled by ImageJ/Micro-Manager (version 1.3). Optical density was determined as described for all quantifications from one region within a worm slice and an adjacent background region.

### Control Reactions for Enzymatic Staining Reactions

To check for unspecific reactivity of the electrophilic dye NBT with *C. elegans* tissues, wild type worms were incubated in NBT solution lacking the substrate. NBT solution (2 ml): 1 ml 0.1% NBT solution, 0.5 ml 0.07 M phosphate buffer, 0.5 ml H_2_O. Sections were incubated until a control reaction (with succinate) turned positive at room temperature (18 min). Both reactions were stopped simultaneously by addition of 4% formalin.

To determine whether to incubate *C. elegans* tissues at room temperature (RT) or at 37°C, a set of wild type slides for SDH were incubated at those two temperatures in parallel. Incubation times were recorded for each temperature and each stain separately until differentiation was strongest. Best differentiation was obtained after 23 min at RT, and after 9 min at 37°C. However, differentiation and staining pattern were more distinct at RT. Complex-specific inhibition was tested by pre-incubation of mounted wild type sections with the respective inhibitor solution. NADH inhibition was achieved in 100 µM rotenone (Sigma Aldrich, Switzerland). SDH inhibition was achieved in 100 mM malonate (Sigma Aldrich, Switzerland). COX inhibition was achieved in 2 mM KCN (Sigma Aldrich, Switzerland). After 30 min incubation with the respective inhibitors, the slides were stained according to the protocols for NADH, SDH, and COX. Uninhibited wild type controls were stained simultaneously and staining was terminated once the controls turned positive with sufficient differentiation (NADH: 8 min., SDH: 14 min., COX: 55 min.)

Inhibitors for different complexes are often used when ETC enzyme activities are measured *in vitro* on isolated mitochondria. Therefore we provided inhibitors directly in the staining solutions to test this approach *in situ*. This led us to realize that inhibitors (or their solvents) tend to markedly lower the pH of the solution, resulting in a major decrease in staining efficiency, thereby precluding their use in additional control reactions.

### Isolation of Mitochondria

*C. elegans* strains *gas-1(fc21), mev-1(kn1)* and N2 were synchronized by hypochlorite treatment and cultivated on NGM plates seeded with *E. coli* OP50 [43]. Worms were collected at day 1 of adulthood with S-basal [48]. 1 g of worm pellet (wet mass) was resuspended in 10 ml of MSE buffer (220 mM mannitol, 70 mM sucrose,10 mM Tris, 2 mM EDTA, pH 7.4). Worms were homogenized with an engine potter (500rpm for 5 times). After homogenization, subtilisin A was added (10 mg/g of worm pellet) and left for 20 min at 28°C, followed by manual homogenization. Subcellular fractionation to isolate mitochondria was performed as follows: the homogenate was spun down at 10'000×g for 5 min at 4°C, resuspended in 10 ml of MSE buffer, 0.4% BSA (fatty acids free), and spun again at 1'000×g for 5 min at 4°C to remove cellular debris. Mitochondria were retrieved from the supernatant by centrifugation at 10'000×g for 5 min at 4°C and resuspended in MSE buffer. Protein concentration was determined using Bradford's method (absorption at 595 nm).

### Spectrophotometric Assays of Individual Respiratory Chain Complexes

All assays were performed at 25°C using a U-3900 spectrophotometer (Hitachi, Japan). All enzyme activities were normalized to protein content.

Complex I activity was measured by following the decrease in absorbance due to oxidation of NADH at 340 nm (ε = 6.22 mM^−1^cm^−1^). The assay was performed in 0.5 ml final volume. Mitochondria (20 µg) were added to buffer containing 50 mM KCL,10 mM Tris-HCL, 1 mM EDTA (pH 7.4), 2 µg/ml antimycin A, 2 mM KCN, 100 µM NADH and 60 µM decylubiquinone (DB) as electron acceptor. The concentration of the DB stock solution was verified at 278 nm (ε = 14.0 mM^−1^cm^−1^). The NADH-ubiquinone oxidoreductase activity was measured for 3 min before addition of rotenone (2 µg/ml) after which the activity was measured for additional 3 min. Complex I activity was expressed as rotenone-sensitive NADH-ubiquinone oxidoreductase activity.

Complex II or succinate-CoQ oxidoreductase activity was measured by following the reduction of the quinone like 2,6-dichloropheno-nolindophenol (DCPIP) at 600 nm (ε = 19.1 mM^−1^cm^−1^). The assay was performed in 1 ml final volume. Mitochondria (20 µg) were added in a buffer containing 50 mM potassium phosphate (pH 7.4), 2 µg/ml rotenone, 2 µg/ml antimycin A, 2 mM KCN, 20 mM succinate, 50 µM DCPIP and afterwards baseline rate was recorded for 3 min. The reaction was started with 65 µM ubiquinone and reduction of DCPIP was measured for 3 min.

Succinate dehydrogenase (SDH) activity was measured as previously described [17]. The assay was performed in 1 ml of 0.1 M Tris-SO_4_ buffer, pH 7.4, containing 0.165 mg phenazine methosulphate (PMS), 0.8 mg cytochrome c, 1 mM potassium cyanide, 20 mM sodium succinate against a reference of 20 µl of 20% sodium malonate. The reaction was started by adding 20 µg mitochondria and activity was measured for 5 min. Cytochrome *c* reduction was measured by the increase in absorption at 550 nm (ε = 21.0 mM^−1^cm^−1^).

Complex IV activity was determined following the oxidation rate of reduced cytochrome *c* at 550 nm (ε = 21.0 mM^−1^cm^−1^). The assay was performed in a final volume of 0.5 ml in 50 mM potassium phosphate (pH 7.4), 2 µg/ml rotenone and 50 µM reduced cytochrome *c.* The reaction started by addition of mitochondria (20 µg) and activity was measured for 3 min. The reaction was inhibited by 2 mM potassium cyanide. Cytochrome *c* was freshly reduced from cytochrome *c* by adding freshly made 0.1 M sodium dithionite, and quickly purified with water on a Sephadex G-25 column (Sigma Aldrich, Germany).

### Photomicrographs

Photomicrographs were taken with a consumer grade digital color camera (Traveler Touch One, Aldi, Germany, 10 Megapixel resolution JPEG output) and mounted routine histology color digital cameras on transmission light microscopes (Zeiss Axioskop, Zeiss Axioplan as well as Olympus). Sensitivity of the camera and light intensity were fixed, exposure times were automatically adjusted and checked for linearity by correlation with background pixel intensity. For each imaging session, the white balance of the cameras was re-adjusted and used for the entire session. No images were directly compared if not taken during the same session from the same slide. A 20x objective was used for quantification of COX, NADH and SDH activities and a 40x objective was used for Oil-Red-O quantification.

### Image Annotation

Image annotation and analysis were performed using ImageJ (version1.43u) [49] and standardized using custom macro functions (available from the authors upon request). Only photomicrographs taken with identical camera settings from sections stained on the same glass slide were directly compared. Images from COX, NADH, and SDH stained sections were annotated upon unequivocal identification of intestine, body wall, pharynx and embryos. As the position within the germline cannot be easily determined on morphological grounds in these three stains, it was not annotated. No distinction was made between different stages of embryogenesis. Instead, embryos were annotated only in longitudinal sections exhibiting rows of eggs within the hermaphrodite. One representative ROI per tissue and animal was annotated, and each embryo was annotated individually. Next to each worm section within a picture, a comprehensive, mostly circular and representative local background area was annotated. These background ROIs close to the sample were used to eliminate any bias derived from unequal specimen illumination. The manual selection process was facilitated by a keyboard shortcut macro in ImageJ which saves ROIs in ImageJ ROI format in one ROI file per image (available from the authors upon request). No distinction between strains was made, and the images were annotated by two observers blinded for strain names. Images of Oil-Red-O stained sections were annotated for 3 tissue types: intestine, distal and proximal germline, including oocytes and intrauterine embryos.

### Quantification of Light Absorption in Enzymatic Histochemistry

The annotated image sets were processed by an ImageJ macro that returns the mean intensity value (8-bit depth) in pairs for each tissue ROI and for its geometrically closest background ROI (available upon request). Optical density was defined as OD  =  log_10_(I_bk_/ I_m_), where I_bk_ denotes background intensity and I_m_ is the ROI intensity [23]. The data were normalized to wild type levels for mutant strains. Confidence intervals and t-test probability values were calculated in OpenOffice (version 3.2, Sun Microsystems).

### Oxygen Consumption Rate Measurements

Oxygen consumption rates were measured using a Oroboros Oxygraph 2k (Oroboros Instruments GmbH, Austria). 300 animals of age day 1 of adulthood were manually picked and transferred to non-seeded NGM plates from where they were immediately recollected with M9 buffer. The suspended worms were transferred to 1.5 ml tubes (Eppendorf, Germany) and washed with M9 buffer. The worms were re-suspended in 50 µl M9 buffer and introduced into the Oxygraph chamber containing 2 ml of M9 buffer, maintained at 20°C. Oxygen consumption was measured for a minimum of 10 min. The slopes of the linear portions of the plots were used to calculate oxygen consumption rates. All measurements were independently performed for at least 3 times. Data were analyzed by DatLab4 software (Version 4.3). Using a t-test in OpenOffice (version 3.2, Sun Microsystems), mutant strains were compared to wild type (N2) animals.

### Image Analysis for Oil-Red-O Staining

Images were automatically processed by a two-step segmentation algorithm in ImageJ. First, background was removed by converting color to gray-scale and applying the “AutoThreshold” function to generate a selection that separates the worm section from the bright background. Second, color segmentation within the selection was performed to distinguish primarily red pixels (Oil-Red-O) from primarily blue pixels (hematoxylin) by the condition: IF red > blue AND red > green THEN consider as red, ELSE IF blue > red AND blue > green THEN consider as blue. Original images and segmented images were visually compared and the algorithm was found to represent the two colors in an unbiased fashion. The Oil-Red-O positivity ratio was defined as PX_OILRED_ / (PX_OILRED_ + PX_HEMATOXYLIN_) where PX_OILRED_ is the number of pixels considered “red” and PX_HEMATOXYLIN_ is the number of pixels considered “blue”. Data series were compared using a t-test in OpenOffice (version 3.2, Sun Microsystems). All three analyzed strains were stained on the same slide and photographed with the identical microscope and camera settings. Image data subsets for figures were selected from the original JPEG files using GIMP (version 2.6, 2010, The GIMP Team). Images in the figures were contrast-enhanced for optimal printing in OpenOffice (version 3.2, Sun Microsystems). All image analyses were performed on unmodified image data only.

## Supporting Information

Figure S1 **Comparison of three different methods for Oil-Red-O staining of neutral fat in** ***C. elegans*** **in whole animals.** (A) Whole-mount permeabilized and Oil-Red-O stained animals on day 1 of adulthood. Note that *lpd-3(ok2138)* animals are thinner than wild type. Staining intensity is not obviously reduced. Blurring results from lipid droplets in various focal planes due to the three-dimensional nature of the specimens. There is an obvious increase in lipid droplets and droplet size in *daf-2(e1370)*. (B) Same sample of animals as in A, embedded in carbowax and sectioned on a cryostat into 7 µm sections. Sections were mounted without further processing on glass slides and covered with aqueous media as described in the methods section. (C) Same sectioning as in B, however sections were counter-stained with hematoxylin as described in the methods section. This would allow for quantification of lipid droplet area in relation to the entire tissue area. Note that morphology is less preserved than in fresh frozen sections.
(PDF)

Figure S2 **Fresh frozen** ***C. elegans*** **sections stained with Oil-Red-O.** Higher resolution images from Figure 4.
(PDF)

Figure S3 **Changes in optical density from ETC enzymatic activities depends on incubation time.** Changes in optical density (OD) plotted over time, after extraction from video recordings of the enzymatic staining for (A) NADH, (B) SDH and (C) COX activity at 20°C. The large difference between reaction times is due to the different incubation times necessary until saturation is reached. Note that all three reactions show an exponential phase during which staining reaction should be stopped for quantification. The beginning of the COX reaction could not be recorded since the slides needed to be sealed with wax prior to mounting on the microscope to prevent a dry-out. Also note that the thickness of the COX plot is a result of more sampling per time and that the different OD range results from the different color of DAB (light brown) compared to NBT (dark blue). All the ETC activities are measured in the intestine of the animal at the day 1 of adulthood.
(PDF)

Figure S4 **Triacylglyceride levels in age-synchronized and mixed-age cultures of N2 and** ***daf-2(e1370)*** **.** Triacylglyceride level was measured by thin-layer chromatography in either synchronized cultures at day 1 of adulthood or mixed-age populations. Bars represent the standard error of the mean. Asterisks indicate statistical significance in comparison to wild type from each group (Student's t-test, *** p<0.001).
(PDF)

Figure S5 **Full-resolution images of frozen and plastic-embedded sections of wild type animals, treated with various staining protocols.** 1. Transversal sections through the mid-body; 2. Diagonal sections through the mid-body; 3. Transversal sections through the head. Staining methods: H&E, toluidine-blue (TOL, plastic), para-phenylene-diamine (PAR, plastic), PAS, enzymatic activities (NADH, SDH, COX), Oil-Red-O (ORO). Diagonal sections through the mid-body (2) allow better visualization of the body wall muscles than transversal sections (1,3). Two groups (dorsal, ventral) of body wall muscles are visible (red arrows) as well as the alae (black arrows) which allow orientation of the sections. The lateral gaps between the muscles are apparent in most stainings, including the enzymatic activities. Other organs (p-pharynx; i-intestinal lumen; g-proximal germline; d-distal germline/oocyte) are recognizable in most stains. Images were taken on a light microscope, 100x oil-immersion objective. Protocols for plastic embedding adapted from Romeis, B. (1989). Mikroskopische Technik (17th ed.). Urban und Schwarzenberg; available on request from the authors.
(PDF)
