# Supplementary figures and images for: A Tissue-Specific Approach to the Analysis of Metabolic Changes in Caenorhabditis elegans
Source: PLoS One. 2011 Dec 5;6(12):e28417. doi: 10.1371/journal.pone.0028417 (PMC3230600; doi:10.1371/journal.pone.0028417)

Figure S1.

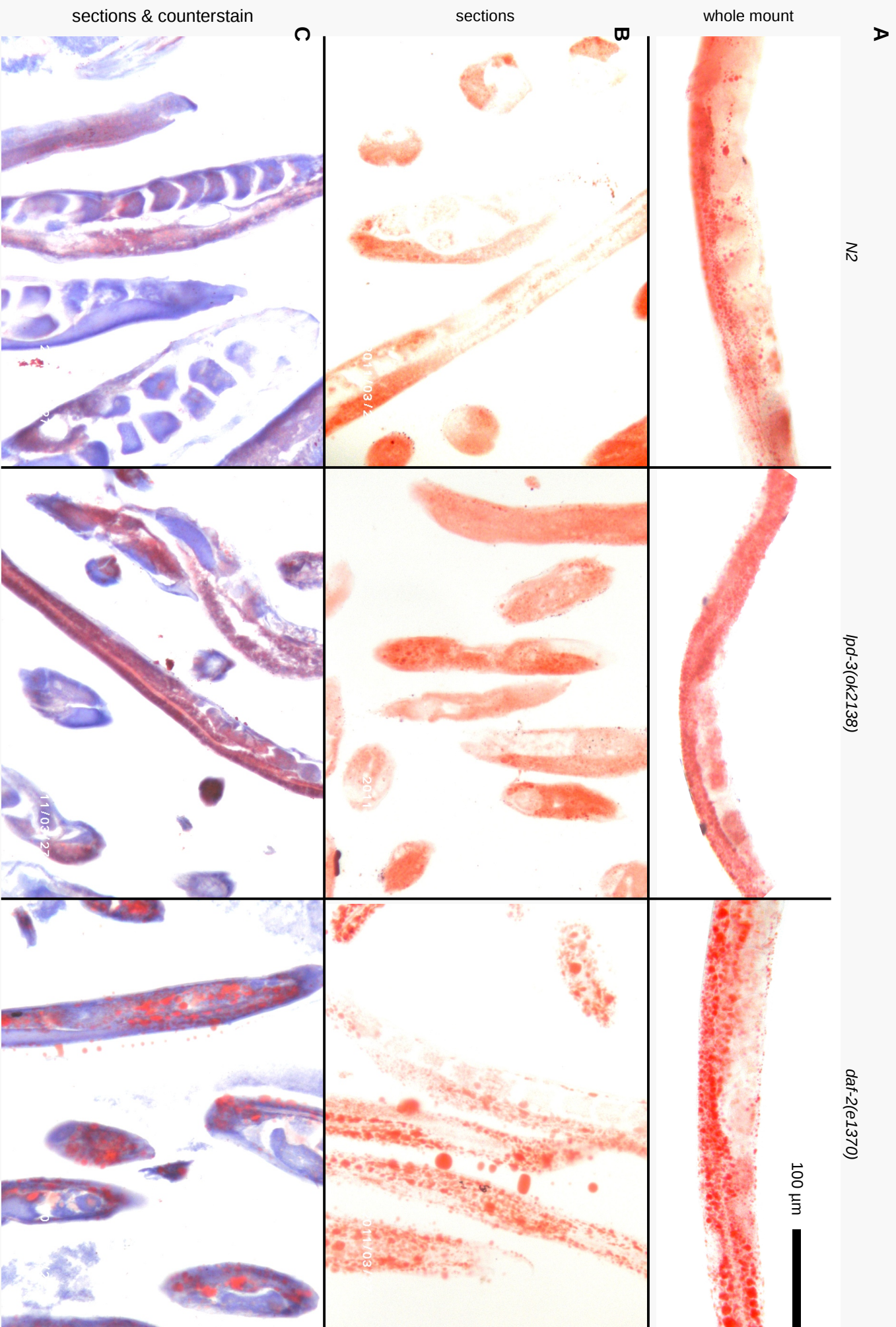

Supplement: Figure S1 — Comparison of three different methods for Oil-Red-O staining of neutral fat in C. elegans in whole animals. (A) Whole-mount permeabilized and Oil-Red-O stained animals on day 1 of adulthood. Note that lpd-3(ok2138) animals are thinner than wild type. Staining intensity is not obviously reduced. Blurring results from lipid droplets in various focal planes due to the three-dimensional nature of the specimens. There is an obvious increase in lipid droplets and droplet size in daf-2(e1370). (B) Same sample of animals as in A, embedded in carbowax and sectioned on a cryostat into 7 µm sections. Sections were mounted without further processing on glass slides and covered with aqueous media as described in the methods section. (C) Same sectioning as in B, however sections were counter-stained with hematoxylin as described in the methods section. This would allow for quantification of lipid droplet area in relation to the entire tissue area. Note that morphology is less preserved than in fresh frozen sections. (PDF) [file pone.0028417.s001.pdf]

Figure S2

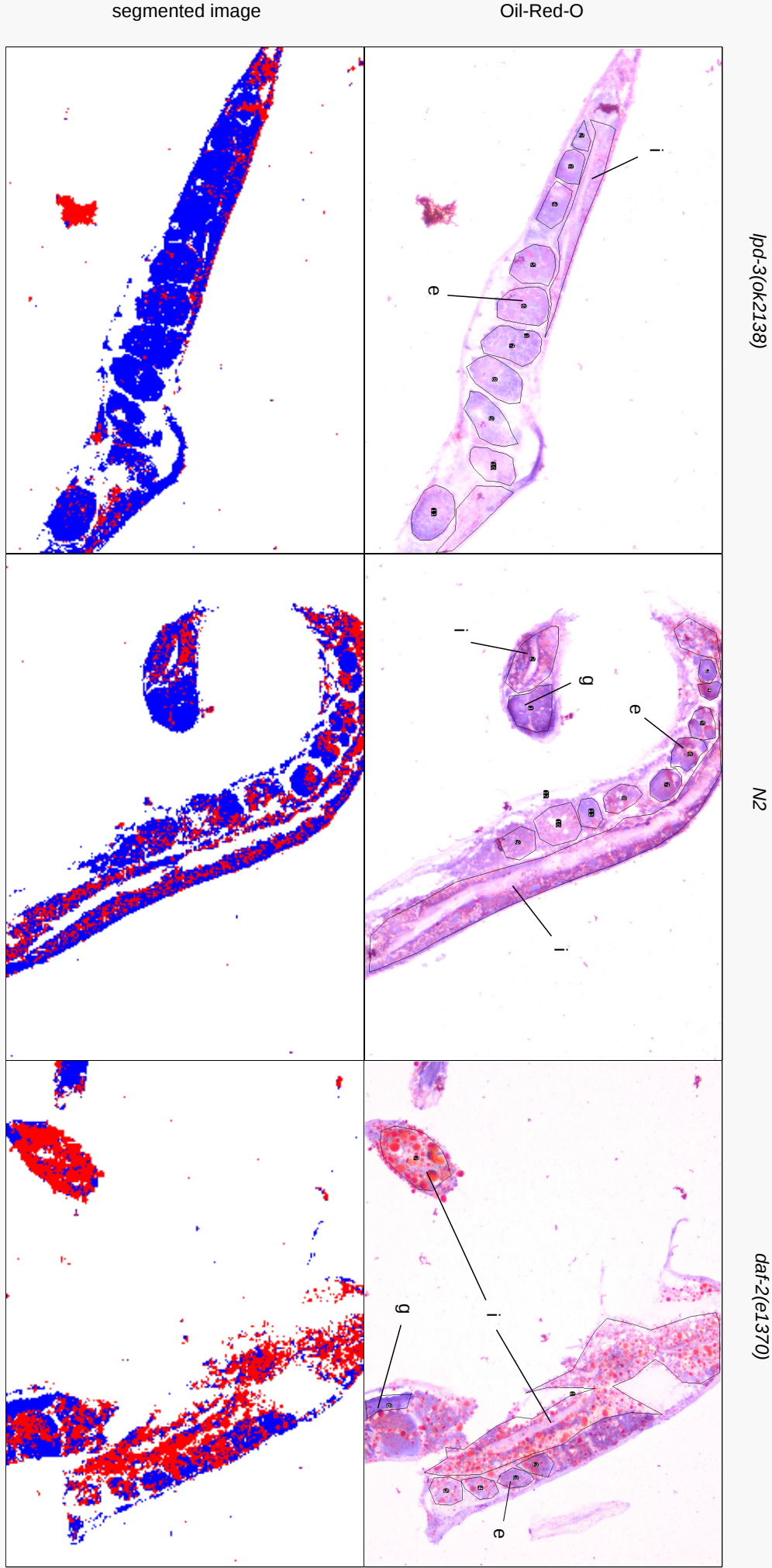

Supplement: Figure S2 — Fresh frozen C. elegans sections stained with Oil-Red-O. Higher resolution images from Figure 4. (PDF) [file pone.0028417.s002.pdf]

Figure S3.

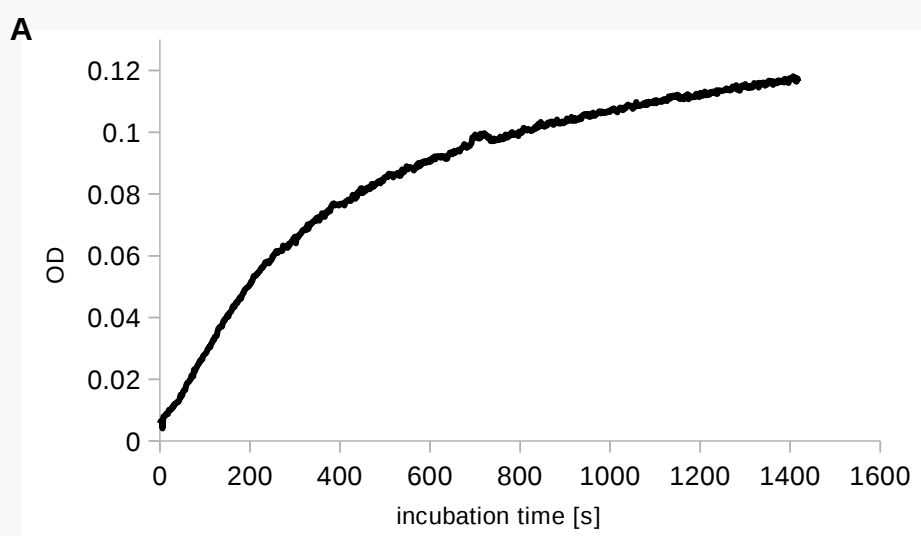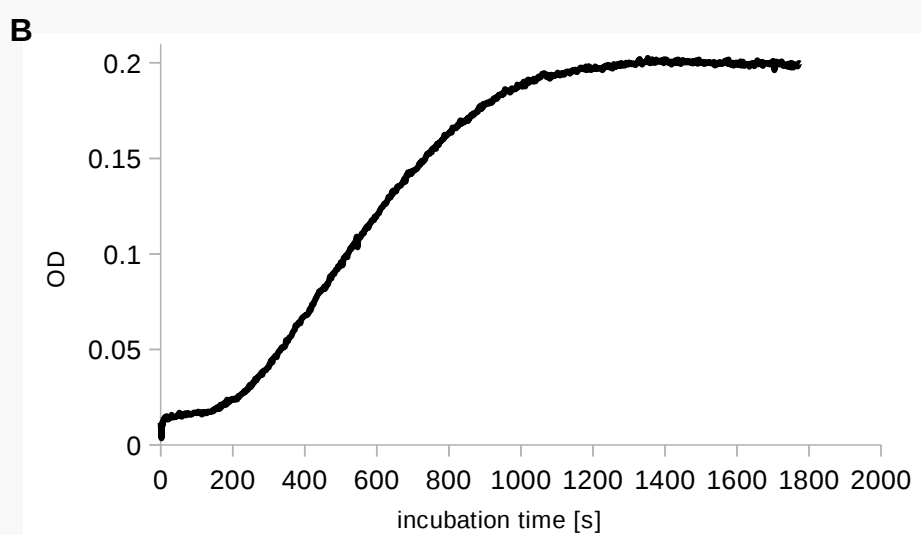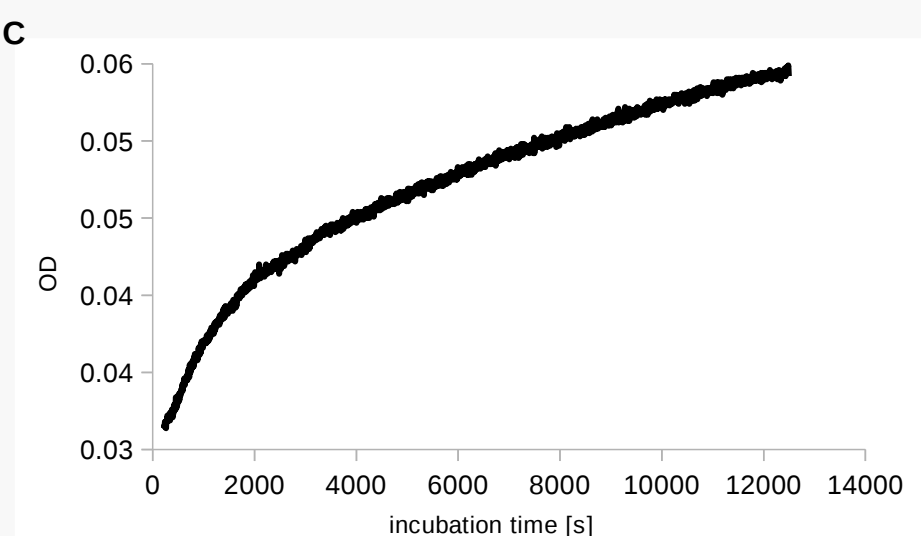

Supplement: Figure S3 — Changes in optical density from ETC enzymatic activities depends on incubation time. Changes in optical density (OD) plotted over time, after extraction from video recordings of the enzymatic staining for (A) NADH, (B) SDH and (C) COX activity at 20°C. The large difference between reaction times is due to the different incubation times necessary until saturation is reached. Note that all three reactions show an exponential phase during which staining reaction should be stopped for quantification. The beginning of the COX reaction could not be recorded since the slides needed to be sealed with wax prior to mounting on the microscope to prevent a dry-out. Also note that the thickness of the COX plot is a result of more sampling per time and that the different OD range results from the different color of DAB (light brown) compared to NBT (dark blue). All the ETC activities are measured in the intestine of the animal at the day 1 of adulthood. (PDF) [file pone.0028417.s003.pdf]

Figure S4.

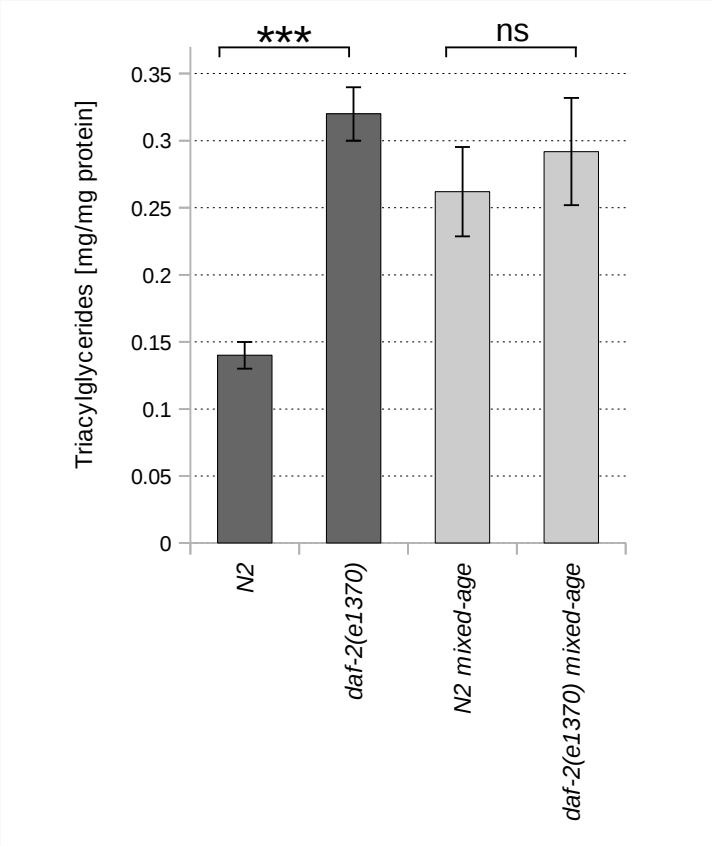

Supplement: Figure S4 — Triacylglyceride levels in age-synchronized and mixed-age cultures of N2 and daf-2(e1370) . Triacylglyceride level was measured by thin-layer chromatography in either synchronized cultures at day 1 of adulthood or mixed-age populations. Bars represent the standard error of the mean. Asterisks indicate statistical significance in comparison to wild type from each group (Student's t-test, *** p<0.001). (PDF) [file pone.0028417.s004.pdf]

Figure S5.

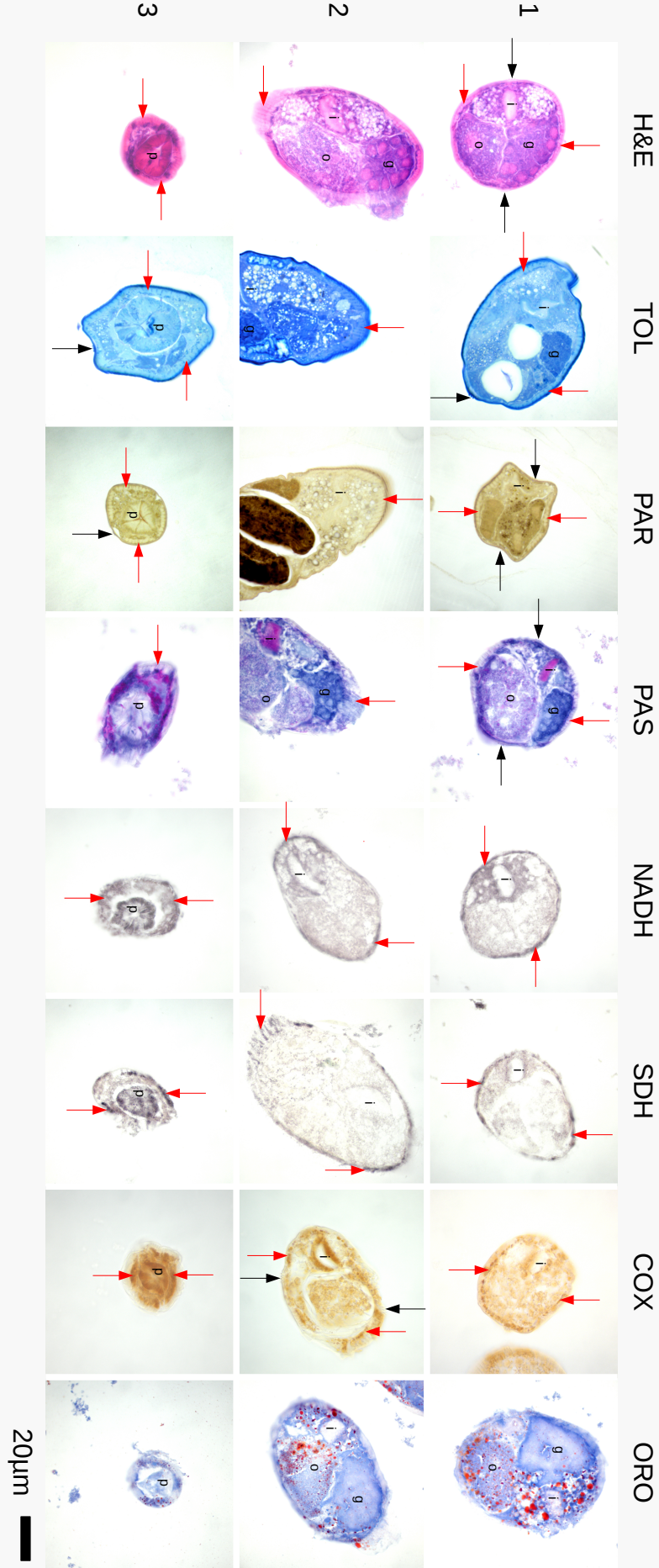

Supplement: Figure S5 — Full-resolution images of frozen and plastic-embedded sections of wild type animals, treated with various staining protocols. 1. Transversal sections through the mid-body; 2. Diagonal sections through the mid-body; 3. Transversal sections through the head. Staining methods: H&E, toluidine-blue (TOL, plastic), para-phenylene-diamine (PAR, plastic), PAS, enzymatic activities (NADH, SDH, COX), Oil-Red-O (ORO). Diagonal sections through the mid-body (2) allow better visualization of the body wall muscles than transversal sections (1,3). Two groups (dorsal, ventral) of body wall muscles are visible (red arrows) as well as the alae (black arrows) which allow orientation of the sections. The lateral gaps between the muscles are apparent in most stainings, including the enzymatic activities. Other organs (p-pharynx; i-intestinal lumen; g-proximal germline; d-distal germline/oocyte) are recognizable in most stains. Images were taken on a light microscope, 100x oil-immersion objective. Protocols for plastic embedding adapted from Romeis, B. (1989). Mikroskopische Technik (17th ed.). Urban und Schwarzenberg; available on request from the authors. (PDF) [file pone.0028417.s005.pdf]
